# Case Report: A Rare Case of Right-Sided Papillary Fibroelastoma in a 1-Year-Old With Congenital Heart Disease

**DOI:** 10.3389/fcvm.2020.624219

**Published:** 2021-01-27

**Authors:** Ali Ahmad, Edward A. El-Am, Reto D. Kurmann, Donald J. Hagler, Melanie C. Bois, Joseph J. Maleszewski, Kyle W. Klarich

**Affiliations:** ^1^Department of Cardiovascular Medicine, Mayo Clinic, Rochester, MN, United States; ^2^Department of Medicine, Indiana University School of Medicine, Indianapolis, IN, United States; ^3^Division of Pediatric Cardiology, Department of Pediatric and Adolescent Medicine, Mayo Clinic, Rochester, MN, United States; ^4^Department of Laboratory Medicine and Pathology, Mayo Clinic, Rochester, MN, United States

**Keywords:** papillary fibroelastoma, cardiac tumor, congenital heart disease, pediatrics, case report

## Abstract

**Introduction:** Cardiac papillary fibroelastomas (PFEs) are the most common primary benign cardiac tumors, although they are somewhat unusual in children and typically seen on the left-sided cardiac valves.

**Case summary:** A 10-week-old patient was found to have a partial atrioventricular canal defect, with associated tricuspid and mitral regurgitation. He was medically managed until 1 year of age, when surgical correction was done. During the procedure, a PFE was found incidentally on the TV.

**Conclusion:** This is one of the youngest patients to be reported with PFE, thus adding to the literature of these unusual cases in children.

## Introduction

Cardiac papillary fibroelastomas (PFEs) are the most common primary benign tumors of the heart (1). Histologically, they consist of avascular fibroelastic tissue surrounded by endocardium (1). While not malignant in the classical sense, they have a clinical importance since they may be a source of systemic embolization due to embolization of either the tumor or adherent surface thrombus (2, 3). Symptoms are frequently the sequelae of embolization and can include angina, shortness of breath, and symptoms of stroke/transient ischemic attack (1–3).

Their etiology is still unknown, though several theories were proposed. Given their propensity to arise on damaged endocardium, it is possible that this injury can result in their formation. In this report, we describe a case of a PFE arising on the tricuspid valve (TV) in a 1-year-old born with congenital heart disease.

## Case Report

A previously healthy prematurely-born (36 4/7 weeks) male presented to his pediatrician at 1-week of age for regular follow up. The pediatrician realized a soft 2/6 systolic murmur with a fixed split. An echocardiogram showed both a partial atrioventricular (AV) canal defect, consisting of a primum atrial septal defect (ASD), and cleft mitral valve (MV). The patient was maintained on medical management (diuretics) and supplemental oxygen for episodic cyanotic spells. At 13 months of age, episodes of persistent cyanosis and respiratory distress occurred, which on work-up revealed pulmonary hypertension. He was then transferred to our institution for definitive surgical correction of his congenital disease.

**Figure 1:**
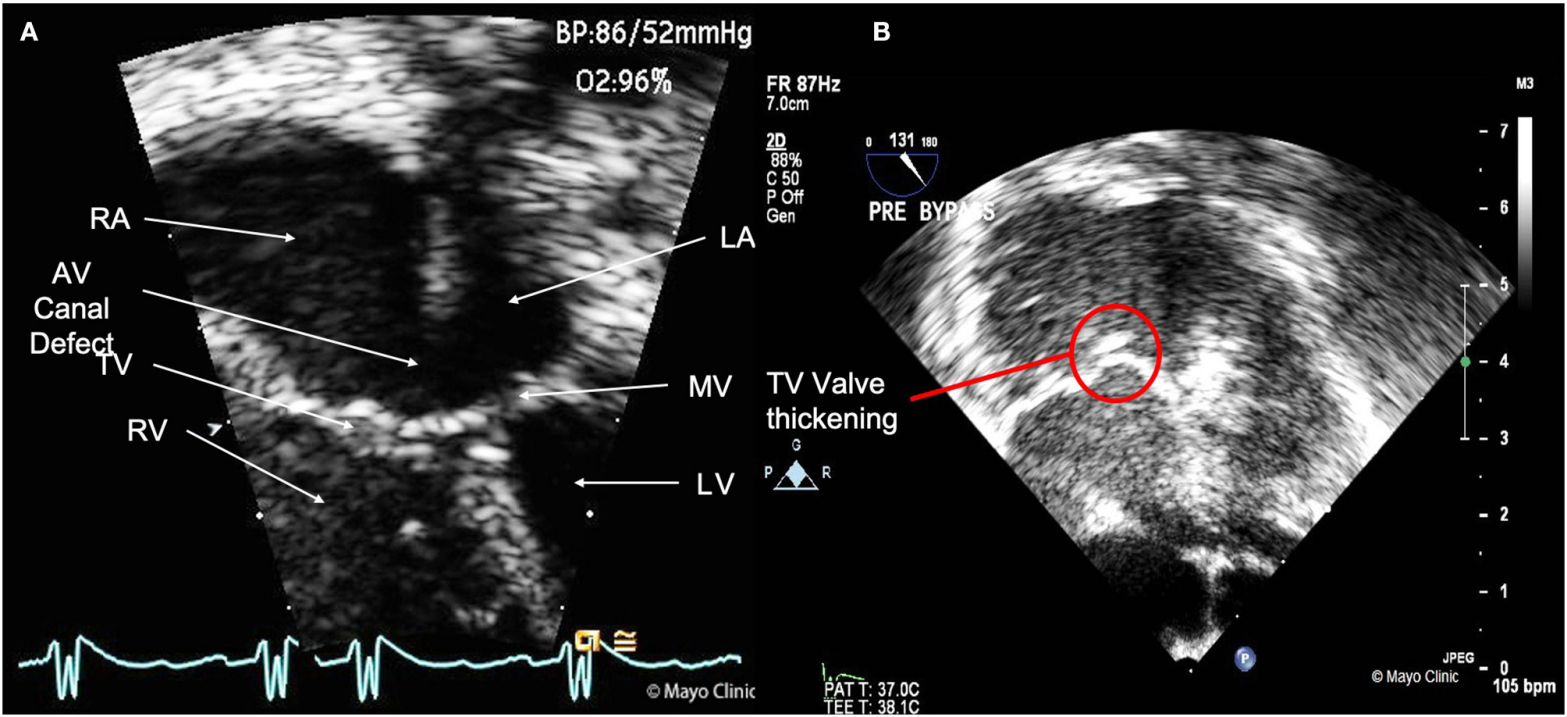
**(A)** Transthoracic echocardiography image showing the 4 cardiac chambers (RA, Right atrium; RV, Right ventricle; LA, left atrium; LV, Left ventricle), mitral valve (MV), tricuspid valve (TV), and the partial AV canal defect. **(B)** Intra-Op transesophageal echocardiography image, with view of the thickened TV.

On admission, the patient was fussy and diaphoretic. Physical exam revealed a grade 3/6 systolic ejection murmur at the upper left sternal border and grade 3/6 diastolic rumble at the left lower sternal border. Chest Xray revealed cardiomegaly, prominent aortic nob and increased pulmonary vascular markings. A detailed preoperative congenital transthoracic echocardiogram reported, in addition to the partial AV canal defect, a thickened MV with moderate-severe regurgitation (Figure 1A, Supplementary Videos 1, 2). Further examination of the MV revealed mild mitral hypoplasia. Left ventricular size and function (ejection fraction = 77%) were normal. The aortic valve was normal. In addition, severe pulmonary hypertension (systolic pulmonary artery pressure = 72 mm Hg) with severe right ventricular and right atrial enlargement with subsequent decrease in right systolic function were noted. Furthermore, thickened TV leaflets with moderate regurgitation was seen, especially during the Intra-Op transesophageal echocardiography (Figure 1B). Detailed findings of the echocardiogram can be found in Table 1. During surgery, a large primum defect and a small secundum defect separated by a muscle bar were noted. The anterior mitral leaflet appeared dysplastic on either side of the cleft with a parachute configuration. The surgeon then partially sutured the mitral cleft and performed commissuroplasty. There was residual mild mitral regurgitation after repair and surgeon could not further intervene on the valve because of concerns that it would make it stenotic. The tricuspid valve was dilated, and a mass was noted. Tricuspid annuloplasty and excision of the mass was done. Finally, the surgeon was able to patch the atrial septal defect (Supplementary Video 3). A 3-mm punch was places in the atrial septal patch given the pulmonary hypertension.

**Table 1:** Detailed pre-op congenital transthoracic echocardiogram.

**Structure**	**Findings**
Right atrium	• Severe enlargement • Partial AV canal defect • Large primum ASD with large left-to-right shunt • Small secundum ASD • Normal inferior vena cava and normal inspiratory collapse
Tricuspid valve	• Moderate regurgitation • Thickened leaflets
Right ventricle	• Mild-moderate decrease in RV systolic function • RV index of myocardial performance = 0.59 • No shunt at ventricular level • Abnormal septal motion
Pulmonary valve and arteries	• Severe pulmonary hypertension
Left atrium	• Normal left atrial size
Mitral valve	• Cleft mitral valve with moderate-severe regurgitation • Annulus = 10 mm • 2 papillary muscles but parachute like
Left ventricle	• Normal size with D-shape. • Normal systolic function • NO LV outlet tract obstruction
Aortic valve and aorta	• Normal valve • No regurgitation • Normal ascending aortic size

**Figure 2:**
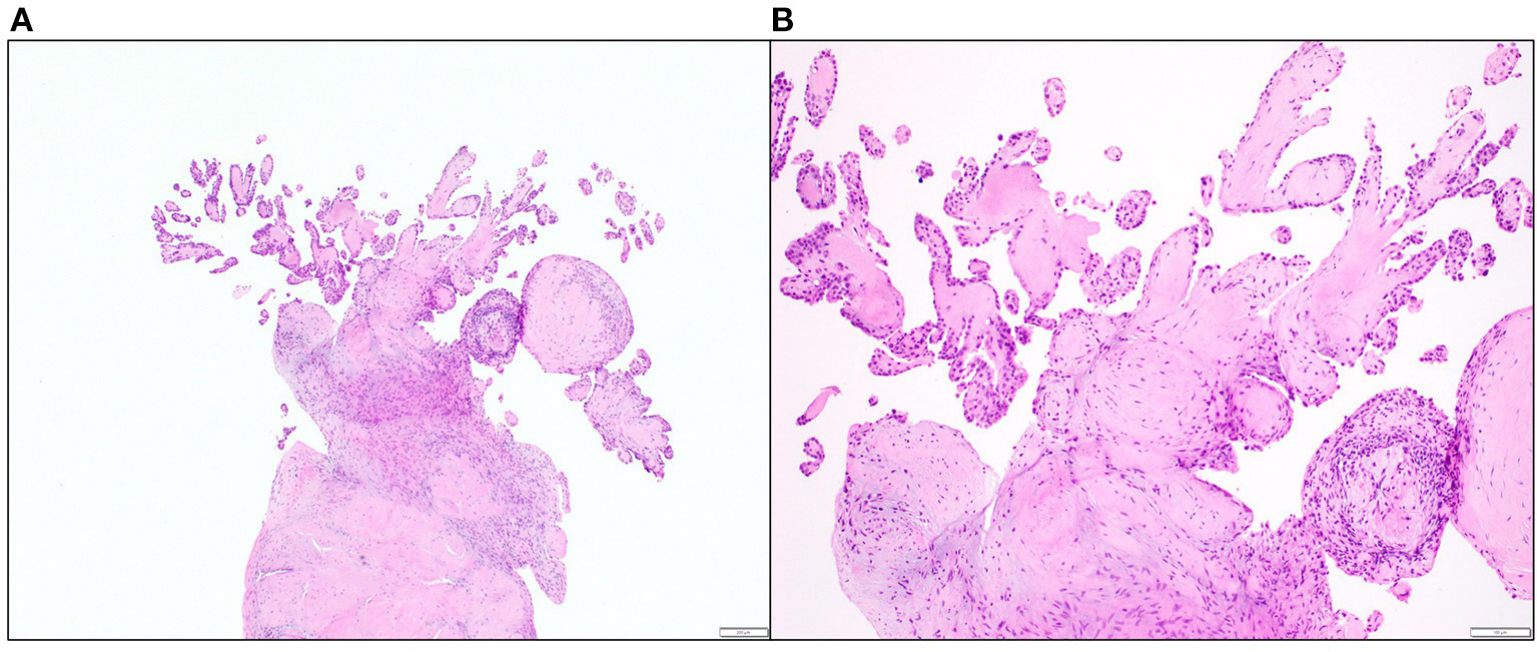
Excised papillary fibroelastoma of a TV leaflet on light microscopic examination **(A)** 40x original magnification, and **(B)** 100x magnification (hematoxylin and eosin stain).

Gross inspection of the TV tissue revealed a 0.7 × 0.2 × 0.2 cm mass that appeared as a portion of pedunculated tan tissue with minute papillary projects. Gross and light microscopic examination confirmed the diagnosis of PFE (Figure 2). Molecular analysis of the specimen did not reveal a *KRAS* mutation on targeted amplification.

The patient had close follow up with regular echocardiograms until 3 years of age. The patient reported no symptoms other than occasional fatigue. Due to severe residual mitral regurgitation, with severe left atrial enlargement (Left atrial volume index = 90 ml/m^2^), he then underwent MV replacement with a mechanical valve. The MV salvaged was found to be dysplastic on pathologic examination, however no PFE was found. Table 2 summarizes the clinical presentation, management and follow up of the patient.

**Table 2:** Summarizes the timeline of events.

At presentation (Age: 10 weeks)	• Murmur on regular physical exam • Echo showed a partial atrioventricular (AV) canal defect, consisting of: • Primum atrial septal defect • Cleft MV
8 months later (Age: 10 months)	• Developed symptoms of heart failure (hypoxia, respiratory distress) • Comprehensive echo showed: • Partial AV canal defect • Thickened MV with moderate-severe regurgitation • Thickened TV with moderate regurgitation • Surgery was done to correct the canal defect and repair the valves (residual MV regurgitation and narrowed orifice) • TV mass seen during surgery was resected. Shown to be papillary fibroeslatoma during pathologic examination
2 years later (Age: 3 years)	• Regular follow up echocardiography showed progression to severe mitral regurgitation and severe left atrial enlargement (no symptoms) • MV replacement was done, no papillary fibroeslatoma shown on pathologic examination

*MV, Mitral valve*.

## Discussion

PFEs are now considered the most common primary cardiac tumor found in the general population (3). The etiology of PFE is not well-established. Multiple theories exist, including the acquired and congenital theories. Not uncommonly, PFEs arising on damaged endocardial surfaces. This postulate is strengthened by multiple observations of PFEs arising on damaged valve. It is also not uncommon in adults for the PFE to be identified in the OR by the surgeon or by intraoperative echocardiography (20%) (3). This highlights the necessity of surgeons and echocardiographers to have a high level of vigilance in patients with underlying endocardial pathology for the possibility of PFE.

Furthermore, most reports in children are in association with congenital heart disease, in which there is abnormal flow and, hence, endocardial injury (4, 5). Two prior cases reported PFE in trisomic patients with cardiac anomalies, although our patient did not have a known chromosomal abnormality (4, 5). More recently, cases of PFE that harbor mutations in the oncogenic driver gene *KRAS* raise the possibility of a subset of these being truly neoplastic – possibly resulting from clonal expansion (possibly after injury) (6). This would essentially suggest the possibility of a complex interplay between environmental factors that potentially result in a genetically driven process.

## Conclusion

This is one of the youngest patients reported in the literature to have a papillary fibroelastoma confirmed by pathology, thus expanding the descriptive spectrum of patients affected by this condition.

## Data Availability Statement

The original contributions generated for the study are included in the article/Supplementary Material, further inquiries can be directed to the corresponding author.

## Ethics Statement

Ethical review and approval was not required for the study on human participants in accordance with the local legislation and institutional requirements. Written informed consent to participate in this study was provided by the participants' legal guardian/next of kin.

## Author Contributions

All authors contributed to the writing and revision of the manuscript.

## Conflict of Interest

The authors declare that the research was conducted in the absence of any commercial or financial relationships that could be construed as a potential conflict of interest.

## Supplementary Material

Supplementary Video 1 Transthoracic echocardiography view of the AV canal defect area. The tricuspid and mitral valves are also visible.

Supplementary Video 2 Transthoracic echocardiography (with Doppler) showing communication through the canal defect and severe mitral and tricuspid regurgitation.

Supplementary Video 3 Post repair transesophageal view of the repaired septal defect.

## Abbreviations

ASD: Atrial septal defect
AV: Atrio-ventricular
AV: Aortic valve
MV: Mitral valve
PFE: Papillary fibroelastoma
TV: Tricuspid valve.
